# The metastatic lymph node microenvironment in adenocarcinomas of the upper gastrointestinal tract: systematic review

**DOI:** 10.1007/s00262-026-04513-w

**Published:** 2026-08-10

**Authors:** John G. Holloway, Ganesh Vigneswaran, Zoë S. Walters, Timothy J. Underwood, Dean Bryant

**Affiliations:** 1 Innovation for Translation Research Group, Faculty of Medicine, School of Cancer Sciences, University of Southampton, Southampton, UK; 2 Faculty of Medicine, School of Cancer Sciences, University of Southampton, Southampton, UK

**Keywords:** Lymph node metastasis, Upper gastrointestinal adenocarcinoma, Tumour microenvironment, Cancer immunology

## Abstract

Lymph node (LN) metastasis in adenocarcinomas of the upper gastrointestinal (GI) tract is a major determinant of patient outcomes following multimodal therapy and surgical resection. Despite its clinical significance, the biology enabling metastatic persistence within lymph nodes remains poorly understood, particularly given the immunologically active nature of the LN microenvironment. This review systematically evaluates the current literature investigating the metastatic LN microenvironment in upper GI adenocarcinomas. A comprehensive search of three databases identified 289 articles, of which 20 met inclusion criteria after screening. Synthesis of the available evidence highlighted findings across cell types within the metastatic LN microenvironment, although B cells and fibroblasts remain comparatively underexplored. Three principal themes emerged from the literature, supporting a hypothesis of dysfunctional anti-tumour immunity within metastatic LNs: macrophages and neutrophils demonstrated pro-tumoural phenotypes characterised by the expression of *SPP1* and *CXCR4*, respectively. Markers of T cell exhaustion were elevated within metastatic LNs although PD-1/PD-L1 expression was lowered, suggesting the use of non-classical immune checkpoint pathways. Current evidence indicates that macrophage- and neutrophil-mediated signalling, including SPP1/CD44 interactions and increased IL-8 expression, may contribute to an immunosuppressive metastatic niche. Collectively, this review consolidates the existing evidence surrounding the metastatic LN microenvironment in upper GI adenocarcinomas and identifies key biological and translational gaps requiring further investigation.

**Supplementary Information:**

The online version contains supplementary material available at https://doi.org/10.1007/s00262-026-04513-w.

## Introduction

Upper GI tract cancers collectively accounted for 1.4 million deaths globally in 2022, mainly consisting of cancers of the oesophagus, gastroesophageal junction, and the stomach [1,2]. Of the two main histological subtypes, adenocarcinomas of the upper gastrointestinal (GI) tract generate a significant burden on healthcare services across the world, but specifically in the western hemisphere [3]. In the current UK clinical setting, the 5-year overall survival is 25% for patients with oesophageal adenocarcinoma (OAC) and 20.9% for patients with gastric adenocarcinoma (GAC) (UK data) [4,5]. However, in patients with nodal metastasis, survival is dramatically reduced; in an Irish cohort, the 5-year survival dropped from 44% (pN0) to 8% (pN3) in patients with resectable OAC [6–8]. Currently, 47% of patients presenting with OAC cannot be treated with curative intent, with the presence of non-regional lymph node (LN) metastases being a major factor in this decision [9,10]. The profound impact of LN metastases on patient outcomes, even after multimodal therapy, underscores the urgent need for a deeper understanding of the underlying disease biology, with the goal of improving outcomes for this patient population with significant unmet clinical need.

LN metastasis is the process by which tumour cells migrate from the primary tumour (PT) and travel through the lymphatic system before infiltrating a tumour draining LN (TDLN) in the regional LN basin. Upon colonisation of the LN, the tumour cells modulate the microenvironment to their benefit through various mechanisms, including both immune reprogramming and stromal remodelling [11]. Furthermore, metastatic tumour cells undergo functional and phenotypic adaptation post-colonisation to survive and expand within the LN microenvironment. As most patients with upper GI adenocarcinomas present with advanced metastatic disease, a greater understanding of the LN microenvironment, the first metastatic site, is of particular importance in this context [12,13]. While reviews examining the metastatic LN microenvironment in other cancers have been published, no review has comprehensively synthesised the available evidence relating to the composition and function of the microenvironment of LN metastases in upper GI adenocarcinomas [14–16]. This review aims to systematically map and synthesise the existing literature examining the cellular composition, activity, and interactions within the metastatic LN microenvironment in adenocarcinomas of the upper GI tract. This review will provide a platform for future research in this field to be conducted with a suitable context and allow for informed, hypothesis-driven research.

## Results

This report follows a systematic approach to ensure comprehensive identification of relevant literature; full methods are provided in the supplementary. Following systematic search and screening, 20 studies met inclusion criteria (Fig. S1, Table S3). Given the methodological and biological heterogeneity of included studies, findings were synthesised around key themes in immune and stromal cell activity within the metastatic lymph node microenvironment.

### Changes in LN immune cell composition

The invasion of metastatic tumour cells is associated with major changes to the immune cell composition of the LN microenvironment. Changes in T cell subset populations are the most commonly reported cell proportion alterations. A reduction in CD8^+^ T cells has been observed in TDLNs compared to the PT, alongside an increased CD4^+^/CD8^+^ ratio in both compartments relative to peripheral blood, supporting the presence of a locally immunosuppressive environment [17–21]. Li et al*.* found that this ratio was further elevated in patients with high serum IL-8 levels (> 100 pg/mL), suggesting a potential modulatory role for IL-8 [18]. The CD4^+^/CD8^+^ ratio alone, however, is difficult to interpret, as variation may reflect changes in either T cell subset independently or simultaneously. Contrary to treatment of naïve metastatic nodes, one study reported increased T cell populations in both the PT and TDLN following neoadjuvant chemotherapy (NAC), though the absence of pre-treatment LN samples limits interpretation [22].

Several studies showed that CD4^+^ T cells were more abundant in the LN than in the PT or distant metastases, with effector memory CD4^+^ cells dominant in TDLNs and enriched in metastatic compared to tumour-free nodes [17,20,21,23]. T helper (Th) cell polarisation was also altered, with a Th1/Th2 skew towards Th2 and enrichment of Th17 signatures in the metastatic LN [21,23]. Additionally, multiple studies observed Tregs as being significantly elevated in metastatic TDLNs compared to metastasis-free nodes, including ICOS^+^ T regulatory (Treg) cells, which positively correlated with plasmacytoid dendritic cells specifically in metastatic nodes, a known mechanism of immunosuppression and poor prognosis in GAC [17,20,23–25]. Increased expression of TGF-β1 in metastatic LNs could explain this change in population of Tregs and CD8^+^ T cells [26].

The size of the B cell population has been reported to vary with changes in the LN microenvironment. A greater proportion of B cells, among immune cells, was observed in the metastatic LN compared to the PT and distant metastases, across studies [27,28]. LN metastases following NAC exhibited significantly higher CD20⁺ B cell density than both pre- and post-treatment PTs [22]. These examples of enrichment may, however, reflect the intrinsic role of LNs as sites of B cell development and should be interpreted with caution when making direct comparisons between LN and PT tissue. Positive correlations were also observed between CD20^+^ B cells, CD8^+^ T cells, and FOXP3^+^ Tregs in post-NAC LN metastases, suggesting coordinated immune infiltration patterns following treatment [22]. These findings clearly highlight the lack of research into B cells and their role in the metastatic LN microenvironment.

The myeloid cell compartment also undergoes metastasis-associated repopulation. Reported in a single study, the CD11c^+^ dendritic cell (DC) proportion was significantly higher in metastatic TDLNs compared to metastasis-free TDLNs [23]. In contrast to the DCs, the total macrophage population was lower in the LN when compared to the PT or distant metastases, even after NAC [21,22]. The cause of this population difference is unclear; however, it is possible that the PT attracts tumour-associated macrophages (TAMs) rather than the metastatic LN excluding resident macrophages, causing this difference. Neutrophil presence, identified by CD16b^+^ staining, was significantly higher in the metastatic LNs compared with the metastasis-free LNs [23]. Elevated IL-8 mRNA and higher expression of the matrix-degrading enzyme MMP2 further supported increased neutrophil recruitment to metastatic nodes [23].

Changes in cell-type proportion provide the first glimpse into how the LN is altered after the arrival of metastatic tumour cells. However, this is often difficult to interpret as using a proportion or ratio metric can be skewed due to further changes in other cell types. Furthermore, comparisons between the immune population of different tissues, especially given the canonically immune-enriched LN environment, provide further challenges to interpreting the comparison. Despite this, there is convincing evidence to suggest a decrease in cytotoxic anti-tumoural immune cells and an increase in a pro-tumoural immune-suppressive population.

### Immune suppression through myeloid reprogramming

Accompanying the evidence supporting the increase in immune-suppressive cellular populations within the metastatic LN microenvironment, cellular-level analysis has revealed phenotypic evidence supporting the notion of immune suppression. One study documented increased surface expression of CD11b and PD-L1 on the metastatic LN DC population suggesting an immune-suppressive role for these cells in the metastatic node [23]. However, despite these cells acquiring immunosuppressive properties, no change in major histocompatibility complex (MHC) class II or CD83 expression was observed, with these cells retaining their antigen-presenting markers [23]. Additionally, the macrophages identified in the LN appear pro-tumoural, evidenced by the activation of the SPP1 pathway in metastatic LNs, supporting the theme of altered tumoural responsiveness [27,28]. Both M1 (anti-tumoural) and M2 (pro-tumoural) polarised TAM subsets have been identified in the metastatic LN; however, M1 subsets were not LN-specific and reportedly difficult to differentiate from the M2 subset by single-cell RNA sequencing (scRNAseq) [28,29]. The LN-resident M2 TAMs interacted with Tregs via the PVR-TIGIT axis, an immune inhibitory pathway [28,30,31]. This Treg involvement is further supported by increased expression of FOXP3 associated with increased metastasis within the LNs [32]. Collectively, this evidence indicates an enrichment of DCs, alongside complex and heterogeneous macrophage activity, with antigen-presenting cells (APCs) broadly exhibiting a shift towards an immunosuppressive phenotype.

Accompanying this altered APC activity, neutrophils were consistently identified to exhibit increased recruitment and functional reprogramming within the TDLN microenvironment. Distinct subclasses of neutrophils have been observed across LNs of differing metastatic status. Two independent studies showed that N0 (naïve) neutrophils expressed high levels of *LYZ*, antimicrobial enzymes, and genes associated with autophagy, phagocytosis, and immune homeostasis, with further enrichment identified for response to lipopolysaccharide and positive regulation of inflammatory response [27,29]. Contrastingly, N2 (pro-tumoural) neutrophils were enriched in metastatic sites and expressed elevated levels of *CXCR4* and reduced levels of CD54, consistent with a pro-angiogenic and pro-invasive phenotype [27,29]. Transcriptomic analyses further demonstrated that N2 neutrophils were enriched for pathways related to cytoplasmic translation, ribosome biogenesis, and oxidative phosphorylation, indicating metabolic reprogramming rather than classical immune activation, further supported by the observation of reduced *HMGB1* and *LCN2* using immunohistochemistry [27]. This suggests that these findings represent the local microenvironment rather than systemic changes. Weighted gene co-expression network analysis (WGCNA) of neutrophil transcriptomes, from scRNAseq data, further correlated *HMGB1* and *LCN2* with modules enriched in neutrophil-mediated immunity, activation, and type I interferon signalling pathways [27], a finding supported by independent single-cell studies highlighting similar pathways in gastric adenocarcinoma metastasis [33].

These findings identify that myeloid cells acquire an immunosuppressive role and are functionally skewed towards a metabolically active, pro-tumoural state, characterised by impaired classical activation alongside increased Treg cell interactions and heightened immune checkpoint signalling. The environment induced here allows for tumour cell growth, and the consequence of this immune suppression is likely to be T cell dysregulation.

### T cell dysregulation

A strong theme throughout the literature is that despite being abundant within TDLNs, T cells display traits of functional dysregulation. Most commonly, CD8^+^ T cells show evidence of exhaustion and dysfunction in the metastatic LN. Multiple studies report a reduced overall CD8^+^ population [17–19], while others describe expansion of cytotoxic and exhausted CD8^+^ subsets during metastatic progression, suggesting that cytotoxic activity may occur alongside functional impairment [18,28]. Exhaustion markers *LAG3,* TIM-3, and PD-1, when co-expressed, have been identified across studies in both the LN and PT, and elevated IFN-γ mRNA expression in TDLNs, with unchanged IL-12 levels, further supports T cell dysregulation [19,23,27]. Consistent with the immune modulatory observations, increased expression of anti-tumour IL-8 receptors (CXCR1 and CXCR2) was also reported more on CD8^+^ cells over CD4^+^ cells in the metastatic LN [18]. A single scRNAseq analysis identified a distinct 20-gene signature in LN-derived exhausted CD8^+^ T cells, replicated across LN and a subset of PT samples, indicating a shared transcriptomic programme across tumour sites [29]. Analogous signatures have been identified in CD4^+^ effector memory, Treg, and exhausted CD4^+^ subsets, though only the CD8^+^ signature has been independently validated [34,35]. During cell interaction analysis, T cells showed the highest number of intracellular communications within the microenvironment, giving further support to the notion in which they are central to the immune organisation [36].

This T cell dysregulation may be partly driven by aberrant immune checkpoint expression. Checkpoint signalling in metastatic LNs appears altered compared to PT, with evidence of both reduced canonical pathway activity and the emergence of alternative regulatory mechanisms. PD-1 expression on T cells has generally been reported as lower in the LNs over the PT [18,19]. Furthermore, the rate of PD-1 expression on all T cells in the non-draining lymph nodes (NDLN) was very low, whereas a separate study found that PD-L1 positivity was detected in 11.8% of distant metastases compared to only 3.6% of metastatic LNs, suggesting potential site-specific variation in expression [18,37]. This raises a question as to what is occurring in the LN that could be actively reducing the expression of PD-1.

Non-canonical immune checkpoint pathways have been observed alongside or instead of PD-1/PD-L1. Limited evidence showed that T cells co-expressing multiple inhibitory immune checkpoints, including PD-1⁺TIM-3⁺, PD-1⁺PD-L1⁺, and TIM-3⁺PD-L1⁺, were significantly enriched in the tumour-infiltrating tissue compared with TDLNs for both CD4^+^ and CD8^+^ T cell population, reinforcing idea of altered checkpoint expression in the LN away from PD-1 [19]. Contrary to regular yet reduced observation of PD-1/PDL-1 expression in metastatic LNs, an alternative single study found that the activation of the PD-1/PDL-1 axis was not detected in the PT or the LN, and the SPP1-CD44 checkpoint expression was instead enriched in the metastatic LN [27]. This observation of an alternative immune-suppression pathway, together with reduced PD-1 expression, provides evidence for a different mechanism of immune evasion in metastatic LNs and highlights the complexity of the biology.

Together, these findings describe a metastatic LN microenvironment in which T cells are abundant but functionally compromised, characterised by CD8^+^ exhaustion, CD4^+^/Treg dominance, and transcriptional reprogramming, all of which likely contribute to the maintenance of metastatic disease. This impairment may be driven through alternative immune checkpoint pathways, potentially sustained by myeloid-mediated immunosuppression within the metastatic LN microenvironment.

### Tumoural reprogramming

scRNAseq analyses have been an important method to gain a greater understanding of the phenotypic differences between the primary and metastatic tumour cells. Pathways, identified in a single report, were enriched in the LN-resident tumour cells and include energy metabolism, protein processing, transport and gene regulation, and necroptosis [36]. Epithelial mesenchymal transition (EMT)-related pathways also were active in tumour cells from the LN, and further analyses highlighted enrichment of necroptosis-related pathways in LN-derived tumour cells with these observations confirmed as tumour-site-specific using spatial transcriptomics [36]. These changes in transcriptional profile allow the tumour to assume a highly active and modular phenotype, primed to adapt to changes within the microenvironment.

It is possible that these phenotypic changes in the tumour cells occur from the influence of other pro-tumoural cells within the microenvironment rather than just a self-reaction to arrival within the LN. MDK–NCL was identified, in a single dataset, as an important LN-specific ligand–receptor pair facilitating communication between epithelial tumour cells and multiple immune cell types, with *MDK* expression enriched in the tumour region and *NCL* predominantly expressed in the stromal compartment [36], showing evidence of paracrine signalling with a tumour-promoting axis within the metastatic node. Further pro-tumoural signalling identified includes a tumour–myeloid signalling axis, LGALS9–P4HB was identified as a communication pathway associated with EMT and lipid metabolism in metastatic cells [21]. Further, tumour-promoting signalling pathways have also been identified and replicated across single-cell datasets. *MIF* signalling from tumour cells to *CXCR4* receptors in B cells and VEGFA-VEGFR1/2 signalling between tumour cells and endothelial cells have been reported in metastatic nodes [27,36]. GAC cells when co-cultured with lymphatic endothelial cells (LECs), key components to LN structure and function, resulted in changes to the immune function of the LECs. LECs exhibited reduced expression of MHC class II molecules yet increased the expression of PD-L1 and indoleamine 2,3-dioxygenase (IDO) [38]. These observations show the tumour cells ability to modify their function and the function of other cells within the metastatic microenvironment to better their chances of survival.

### Stromal-driven environmental remodelling

The stromal cell population also has a major role in generating a tumour-permissive environment within the metastatic LN. Metastatic TDLNs were seen to be abundant with activated fibroblasts similar to PTs, whereas NDLNs and met-free TDLNs have considerably few activated fibroblasts [18]. Implying that the presence of the tumour cells is essential for the remodelling, IL-8 is thought to be the activating factor for these fibroblasts in metastatic LNs. Endothelial cells, fibroblasts, and myeloid cells exhibited the highest number of ingoing and outgoing signalling interactions with epithelial cells further supporting this [21]. Alongside fibroblasts, endothelial cells have a distinct role in generating this tumour-permissive environment. scRNAseq analysis identified a distinct endothelial cell subpopulation present exclusively at metastatic sites, including LN and ovarian metastases [29]. These endothelial cells were characterised by increased expression of angiogenic and lymphangiogenic receptors (*VEGFR* and *FGFR1*) and communicated with pro-angiogenic tumour populations through VEGFA–FLT1 signalling. Heightened expression of the marker genes for this endothelial population was significantly associated with worse overall survival, using the TCGA GAC cohort [39,40]. Endothelial cells were thought to be further modified in metastatic LNs with low TSP1 and high VEGF expression further promoting angiogenesis, although VEGF expression was not correlated with microvessel density [33].

Alongside cellular changes, architectural remodelling of the metastatic LN microenvironment is also a distinct characteristic of metastatic invasion. In metastatic LNs, cartilage oligomeric matrix protein (COMP) expression was reported to be reduced, which was associated with decreased immune infiltration and denser collagen fibres [41]. With further ECM remodelling being contributed through increased ADAMST1 immunoreactivity in epithelial cells of metastatic LNs [33], metastatic LNs further exhibited distinct patterns of stromal remodelling, with fibrosis strongly associated with advanced disease and poorer clinical outcomes. Tumour and stromal regions in the PT and LN have been reported in multiple studies to be morphologically distinct from one another [36,42]. In LNs, fibrosis associated with metastatic tumour cells is frequently accompanied by disruption of the nodal capsule and destruction of normal nodal architecture, indicating substantial structural remodelling [42]. Contrastingly, metastases in LNs without fibrosis were often seen to be restricted to the lymphatic channels surrounding the LN capsule in conjunction with a preservation of the nodal architecture. Patients with a higher proportion of resected nodes exhibiting fibrosis were associated with higher T and N staging, higher American Joint Committee on Cancer stage, the presence of lymphatic invasion, and the presence of venous invasion [42]. These patients also showed significantly shorter overall and disease-free survival. These observations clearly show the impact of stromal architecture and fibrosis on the biology of the node and ultimately outcomes for the patients affected.

These results show the importance of the stromal cell population in generating a tumour-supportive environment and emphasise the stromal cells’ role in direct communication with metastatic tumour cells but also to regulate the non-cellular factors within the microenvironment to support tumour cell proliferation.

## Discussion

This review synthesises the current evidence describing the biological and immunological landscape of LN metastasis in upper GI adenocarcinomas. Across studies, metastatic lymph nodes represented an immunologically distinct niche characterised by functional immune suppression driven by coordinated T cell dysregulation and myeloid reprogramming, rather than classical immune checkpoint activation. LNs were consistently shown to represent a distinct and dynamically remodelled microenvironment rather than an extension of the PT. There was little discourse in the biological evidence surrounding this topic, although this could well be a reflection on the minimal number of studies published on this topic to date.

Despite their continued presence, CD8^+^ T cells appear to be functionally impaired in the metastatic LN microenvironment, suggesting a state of ineffective anti-tumour immunity. Emerging evidence indicates that this process of accelerated exhaustion may be regulated through components of the microenvironment, specifically cytokine signalling and interactions with myeloid cell populations. In particular, IL-8 has been implicated in promoting CD8⁺ T cell dysfunction through the IL-8 signalling axis driven by macrophages. A study of colorectal cancer PTs identified IL-8 to CXCR2 signalling as the key driver of T cell dysregulation [43]. A further study of GAC PTs highlighted macrophage-secreted IL-8 drives immunosuppression of CD8^+^ T cells through reduced tumour infiltration resulting in poor clinical outcomes [44]. This supports the concept, highlighted in this review, of a pro-tumoural shift in myeloid cell activity within metastatic LNs, whereby myeloid populations contribute directly to the suppression of cytotoxic T cell function. Which goes on to suggest that CD8⁺ T cell dysfunction in the metastatic LN microenvironment arises from complex cellular interactions rather than intrinsic exhaustion alone.

Myeloid cells, particularly neutrophils and macrophages, are reprogrammed towards immunosuppressive and tumour-supportive states. Macrophages and neutrophils have both been implicated to be residing in a pro-tumoural state within the metastatic LN microenvironment. Published evidence suggests that tumour-associated myeloid cells and myeloid-derived suppressor cells are recruited from the peripheral bloodstream during the formation of the pre-metastatic niche, which may partly explain their capacity to directly influence tumour cell behaviour, rather than being solely shaped by tumour-derived signals [45,46]. In OAC and gastroesophageal junctional adenocarcinoma, increased pro-tumoural myeloid cell infiltration in the PT is associated with increased nodal metastasis, suggesting a varying role in the acquisition of metastatic potential alongside their importance in founding the nodal metastatic colony reported in this study [47,48]. Additionally, across cancer types, myeloid cells are reported as essential mediators of metastatic infiltration within LNs [49]. Collectively, these findings support a model in which myeloid cells act as central regulators of the metastatic niche, coordinating both immune suppression and tumour-supportive signalling through interconnected pathways.

**Fig. 1:**
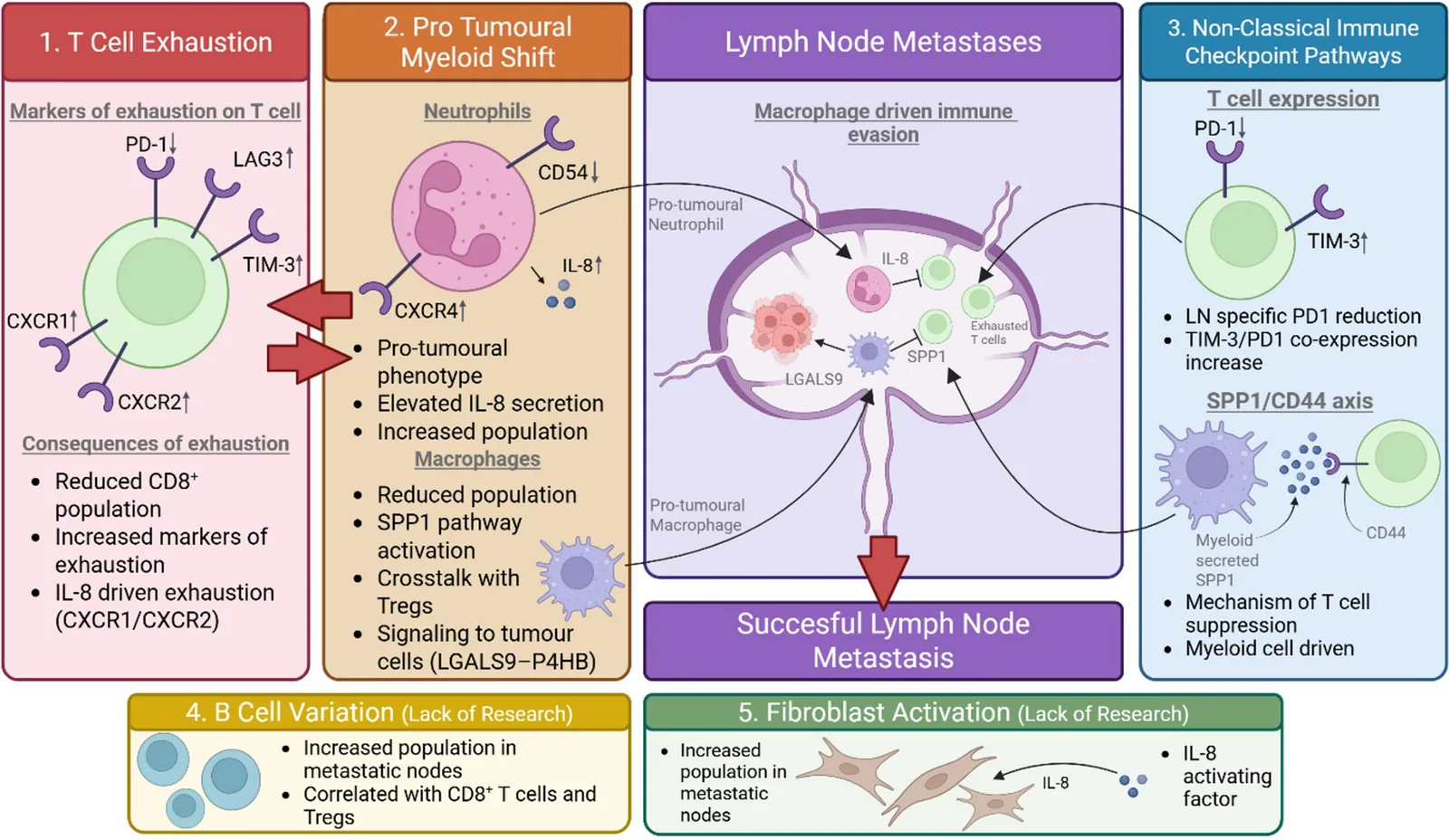
Immune suppression and myeloid reprogramming drive lymph node (LN) Metastasis. This schematic illustrates the relationship between immune mechanisms that promote tumour progression and LN metastasis. (1) T cell exhaustion is characterised by increased expression of inhibitory receptors (LAG3, TIM-3), decreased expression of PD-1 and altered chemokine receptor signalling (CXCR1/CXCR2), resulting in reduced CD8⁺ T cell function and IL-8-driven dysfunction. (2) A pro-tumoural myeloid shift is marked by the increase in neutrophil population (CXCR4^high^, CD54^low^) and increased IL-8 secretion, alongside macrophage alterations including reduced abundance, activation of the SPP1 pathway, and signalling towards regulatory T cells (Tregs) and tumour cells (3). Non-classical immune checkpoint pathways further suppress T cell activity, including LN-specific modulation of PD-1, co-expression of TIM-3/PD-1, and signalling through the SPP1–CD44 axis driven by myeloid cells. Within the LN, macrophage-mediated tumour immune evasion and interactions between neutrophils, macrophages, and exhausted T cells create a permissive niche for metastasis, involving factors such as IL-8, SPP1, and LGALS9. Together, these processes facilitate immune escape and enable successful lymph node metastasis. B cells (4) and fibroblasts (5) both exhibit changes in population within the metastatic LN microenvironment although a current lack of research in this area prevents the exact role of these cell types being determined. Created in BioRender. Holloway, J. (2026)

Multiple studies have reported that PD-1 expression is significantly lower in the LNs compared to the PT, irrespective of their tumour draining status and PD-1 expression increases in the distant metastasis. This suggests the change in immune evasion mechanism is specific to the metastatic LN. Given this, it is likely that the metastatic tumour is utilising an alternative pathway of immune evasion, which appears to be a biological characteristic of metastatic tumours of adenocarcinomas of the upper GI tract. Various studies across cancer types have detected a higher rate of PD-1 expression in in the TDLN compared to the PT [50,51]. Consequentially, this shows inconsistencies between biological activity across cancer types and that sweeping generalised biological concepts are not necessarily applicable to all tumour types. This observation of reduced PD-1 expression further strengthens the argument for an alternative method of immune suppression within the metastatic LN microenvironment and that checkpoint blockade alone may not fully explain immune escape in LN metastases. These programmes of immune activity and evasion all synergise to form a comprehensive mechanism of tumour proliferation within the metastatic LN microenvironment (Fig. 1).

Despite their potential importance in shaping immune organisation and signalling within lymph nodes, fibroblasts and B cells remain comparatively under-characterised. It is currently unclear whether these cells are passive or particularly pro/anti-tumoural, meaning that one of the largest immune cell populations within the LN is not well understood in a metastatic context. Infiltrating B cells and tertiary lymphoid structures at the site of PTs have been associated with improved response to immune therapy, an understanding of this in the metastatic tumour could identify therapeutic opportunities [52,53]. Furthermore, given their abundance within the LN microenvironment and their emerging roles in facilitating metastatic progression, additional focused investigation into LN fibroblast reprogramming is warranted.

Interpretation of these findings must be considered alongside attention to potential limitations. The complexity of the search term and the variations in spelling and nomenclature will potentially have omitted papers from the search results. This was mitigated by the incorporation of all spelling forms of oesophageal and esophageal through using the subject mapping function in EMBASE and MEDLINE. Currently, there is no widespread consensus on the nomenclature describing metastatic or TDLNs, and these terms are often not well defined in each study. This makes it difficult to determine the exact state of each LN being described and how to interpret the biology. The search identified studies with a strong GAC skew, with only 5 studies of the 20 analysing samples collected from patients with OAC. This reduces the generalisability of the observations in this review, but it does reaffirm the importance for further research in the field of OAC. Finally, given the low number of papers identified during the literature search, most of the observations have not yet been replicated and therefore should be interpreted hesitantly until validated in a larger independent cohort.

Collectively, these findings highlight the metastatic lymph node as a complex, heterogeneous immune organ substantially remodelled by metastatic infiltration. CD8^+^ T cell exhaustion and myeloid-driven reprogramming emerge as key mediators of this process. While immune evasion, pro-tumoural immune recruitment, and tumour phenotypic plasticity remain central to metastatic biology, this review provides greater mechanistic context underlying these processes. This review has systematically collated and synthesised the current landscape of research in this field and highlighted emerging insights into the LN microenvironment while also identifying methodological strengths and ongoing challenges. The current body of scientific evidence has underscored the complexity and translational potential of studying the metastatic LN. Continued research in this area is likely to develop a greater understanding of the metastatic microenvironment in these cancers and in metastatic LNs in a pan-cancer context, with the ultimate aim to aid in the development of more effective therapeutic strategies.

## Supplementary Information

Below is the link to the electronic supplementary material.

Supplementary file1 (DOCX 92 kb)

## Data Availability

No datasets were generated or analysed during the current study.

## Abbreviations

APC: Antigen-presenting cell
COMP: Cartilage oligomeric matrix protein
DC: Dendritic cell
EMT: Epithelial mesenchymal transition
GAC: Gastric adenocarcinoma
GI: Gastrointestinal
LEC: Lymphatic endothelial cells
LN: Lymph node
MHC: Major histocompatibility complex
NAC: Neoadjuvant chemotherapy
NDLN: Non-draining lymph node
OAC: Oesophageal adenocarcinoma
PT: Primary tumour
scRNAseq: Single-cell RNA sequencing
TAM: Tumour-associated macrophage
TDLN: Tumour draining lymph node
Th: T helper
Treg: T regulatory
