# Supplementary material for: The metastatic lymph node microenvironment in adenocarcinomas of the upper gastrointestinal tract: systematic review
Source: Cancer Immunol Immunother. 2026 Aug 10;75(11):272. doi: 10.1007/s00262-026-04513-w (PMC13748182; doi:10.1007/s00262-026-04513-w)
Supplement: Supplementary file 1 [file 262_2026_4513_MOESM1_ESM.docx]

**Supplementary Materials**

**Cancer Immunology, Immunotherapy (submitted 2026) – John Holloway *et al.***

**Methods**

Prior to the commencement of the systematic review this project was registered with the Open Science Framework (OSF: [10.17605/OSF.IO/A8MDH](https://doi.org/10.17605/OSF.IO/A8MDH)) and was conducted in accordance with the Preferred Reporting Items for Systematic reviews and Meta-Analyses (PRISMA) statement guidelines.

*Search Strategy*

Three databases, EMBASE (OVID), MEDLINE (OVID) and Web of Science, were electronically searched on 17/12/2025. Both MeSH and free text terms were used to identify papers concerning LN metastasis in adenocarcinomas of the upper GI tract (Table 1). This review included OAC, GAC and gastroesophageal junctional adenocarcinoma. Oropharyngeal adenocarcinoma was not included in this study due to its rarity and differing biology. Key themes included in the search were adenocarcinomas of the upper GI tract, LNs, metastasis and the tumour microenvironment. All articles were extracted into EndNote and downstream analysis was conducted using the Rayyan systematic review organisation software [1, 2]. Rayyan software was used to identify duplicate papers, and duplications were verified manually and removed. The main author (JH) conducted title and abstract screening followed by full text screening, a second independent reviewer (DB) screened a random subset of articles at both points (20% of articles screened), as suggested in the Cochrane handbook [3]. Conflict resolution was not necessary.

*Supplementary* *Table 1: Table of search terms and databases used to identify relevant research papers*

| Database | Search Term |
| --- | --- |
| EMBASE | 1. esophageal adenocarcinoma/  2. stomach adenocarcinoma/  3. (gastroesophageal junction adenocarcinoma or esophagogastric junction adenocarcinoma).ti,ab,kw.  4. (gastric adj3 adenocarcinoma).ti,ab,kw.  5. gastric cancer.mp.  6. 1 or 2 or 3 or 4 or 5  7. exp lymph node/  8. (lymph* adj2 node).ti,ab,kw.  9. 7 or 8  10. tumour microenvironment.ti,ab,kw.  11. tumor microenvironment/  12. 10 or 11  13. esophagus metastasis/  14. exp lymph node metastasis/  15. gastric metastasis/  16. 13 or 14 or 15  17. 6 and 9 and 12 and 16 |
| MEDLINE | 1. Esophageal Neoplasms/ AND Adenocarcinoma/   2. Stomach Neoplasms/ AND Adenocarcinoma/   3. (gastroesophageal junction adenocarcinoma or esophagogastric junction adenocarcinoma).ti,ab,kw.   4. (gastric adj3 adenocarcinoma).ti,ab,kw.   5. gastric cancer.mp.   6. 1 or 2 or 3 or 4 or 5   7. Esophageal Neoplasms/ AND Neoplasm Metastasis/   8. exp Lymph nodes/   9. (lymph* adj2 node).ti,ab,kw.   10. 8 or 9   11. tumour microenvironment.ti,ab,kw.   12. Tumor Microenvironment/   13. 11 or 12   14. Lymphatic Metastasis/   15. Stomach Neoplasms/ AND Neoplasm Metastasis/   16. 7 or 14 or 15   17. 6 and 10 and 13 and 16 |
| Web of Science | 1. TS=("esophageal adenocarcinoma" OR "oesophageal adenocarcinoma")  OR TS=("gastric adenocarcinoma" OR "stomach adenocarcinoma")  OR TS=("gastroesophageal junction adenocarcinoma"  OR "esophagogastric junction adenocarcinoma"  OR "oesophagogastric junction adenocarcinoma")  OR TS=("gastric* adenocarcinoma")  OR TS=("gastric cancer")  2. TS=("lymph node*" OR "lymph nodes")  3. TS=("tumor microenvironment" OR "tumour microenvironment")  4. TS=("lymph node metastasis" OR "lymph node metastases"  OR "gastric metastasis" OR "gastric metastases"  OR "esophageal metastasis" OR "oesophageal metastasis")  5. #4 AND #3 AND #2 AND #1 |

*Inclusion and exclusion criteria*

Articles were included if they fulfilled the following criteria: Original research studies using patient-derived samples (e.g., LN tissue, PT tissue, blood, or other biospecimens relevant to metastasis). Studies involving adenocarcinomas of the lower oesophagus, gastroesophageal junction, or proximal stomach, where LN biology or microenvironment was investigated. Full-text articles published in peer-reviewed journals. Studies published in English. Similarly, the articles were excluded according to the following criteria: Studies investigating cancer types other than oesophageal, gastroesophageal junction, or gastric adenocarcinomas (e.g., squamous cell carcinoma). Studies based solely on animal models, cell lines, or organoids, without patient-derived validation. Review articles, conference abstracts/proceedings, editorials, commentaries, or secondary research. Articles for which full text is unavailable in English.

*Supplementary* *Table 2: Table of inclusion and exclusion criteria using the PECOS framework. Population, Exposure, Comparator, Outcome, Study Design*

|  | Inclusion | Exclusion | |
| --- | --- | --- | --- |
| **Population** | Patient derived samples  Samples from patients diagnosed with oesophageal adenocarcinoma, gastric adenocarcinoma, oesophagogastric adenocarcinoma | Studies investigating cancer types other than oesophageal, gastroesophageal junction or gastric. Studies investigating cancer histologies other than adenocarcinomas (e.g., squamous cell carcinoma). Studies based solely on animal models, cell lines, or organoids, without patient-derived validation. | |
| **Exposure** | Any profiling or characterisation of the metastatic lymph node microenvironment | Any profiling or characterisation of only the primary tumour microenvironment | |
| **Comparator** | No comparator | No exclusions based on comparator | |
| **Outcome** | Cell type composition, immune infiltration, stromal organisation, transcriptional state, or cell-cell communication within the metastatic lymph node microenvironment | No exclusions based on outcome measure | |
| **Study Design** | Full text article  Published in peer review journal  Published in English | Review articles  Conference articles/proceedings  Editorials  Commentaries  Secondary research | |

*Data Extraction and Analysis*

Alongside standard bibliographic information, key themes of results and observations were identified for extraction to align to the aims and objectives of this review article. In addition, information on methodological approaches was collected as well as information on the biology directly pertaining to the metastatic LN microenvironment. Data extraction was carried out by a single reviewer (JH) and results were collected using the Rayyan software before being exported to a csv file. Meta-analysis was not appropriate due to the substantial heterogeneity in study design, tumour type, analytical methods, and reported outcomes.

**Results**

The database search identified a total of 289 records across three databases: EMBASE (n = 139), MEDLINE (n = 41), and Web of Science (n = 109) (Figure S1). Following import into the Rayyan screening tool, 71 duplicate records were removed. One instance of erroneous duplicate identification was detected during manual verification of the automatically identified duplicates. Post duplicate removal, 218 records remained and were screened based on titles and abstracts. Of these, 187 records were excluded as they did not meet the inclusion criteria. The remaining 31 articles were retrieved for full-text assessment and underwent a second stage of screening. Full-text articles were excluded for the following reasons: non-English language (n = 1), conference proceedings (n = 2), inappropriate biological sample (n = 4), and incorrect study design (n = 2). A total of 20 studies met the inclusion criteria and were included in the final data extraction and synthesis for this systematic review (Table S3). During abstract screening two articles were rejected by the second reviewer that were kept by the first reviewer. However, no formal conflict resolution was required, as the discrepant articles were independently excluded at full-text screening.

*Fig S1: PRISMA flow diagram of excluded and included articles. Figure was created using the PRISMA template 2020 [4]*

Of the 20 included studies, all were peer-reviewed journal articles reporting primary research. Publication dates ranged from 1999 to 2025, with most studies published within the past decade (n = 17). All studies analysed patient-derived LN samples from individuals with adenocarcinomas of the upper GI tract. Most studies focused solely on GAC as the PT type (n = 15), whilst a smaller number included cases of OAC or gastroesophageal junctional adenocarcinoma (n = 2). Several studies investigated both GAC and OAC, accounting for the overlap between tumour types (n = 3). Methodologically, the studies could be broadly categorised into predominantly omics-based analyses (n = 6) and wet-laboratory experimental analyses (n = 14). The majority employed *in vitro* and/or *ex vivo* techniques applied to patient-derived material, including flow cytometry and immunohistochemistry. A smaller number of studies utilised in silico bioinformatic approaches for data analysis.

*Supplementary* *Table 3: Table identified studies after systematic search and screening. Paper marked with a * satisfied all screening criteria but did not provide suitable biological results for inclusion within this review.*

| **First Author** | **Year** | **Title** | **Reference in full text** | **Journal** |
| --- | --- | --- | --- | --- |
| **Hu YH** | 2025 | Single-cell and spatial dissection of necroptosis spatiotemporal evolution driving lymph node metastasis in gastric cancer | [36] | Cell Death Discovery |
| **Wang LH** | 2010 | Correlation between elevated FOXP3 expression and increased lymph node metastasis of gastric cancer | [32] | Chinese Medical Journal |
| **Hong SA** | 2018 | Prognostic value of fibrosis ratio in metastatic lymph nodes of node-positive advanced gastric cancer | [42] | Medicine |
| **Tokumoto M** | 2017 | Immunoregulatory Function of Lymphatic Endothelial Cells in Tumor-draining Lymph Nodes of Human Gastric Cancer | [38] | Anticancer Research |
| **Maehara Y** | 1999 | Role of transforming growth factor-β1 in invasion and metastasis in gastric carcinoma | [26] | Journal of Clinical Oncology |
| **Zhang YP** | 2025 | Single-cell transcriptomics reveals the multidimensional dynamic heterogeneity from primary to metastatic gastric cancer | [28] | Iscience |
| **Svensson MC** | 2022 | High Infiltration of CD68+/CD163- Macrophages Is an Adverse Prognostic Factor after Neoadjuvant Chemotherapy in Esophageal and Gastric Adenocarcinoma | * | Journal of Innate Immunity |
| **Okita Y** | 2015 | Alteration of CD4 T cell subsets in metastatic lymph nodes of human gastric cancer | [23] | Oncology Reports |
| **Chen J** | 2013 | Significance of ADAMTS1 expression in gastric cancer and lymph node with metastasis | [33] | Digestive Diseases and Sciences |
| **Zhu X** | 2025 | Myeloid-Derived LGALS9-P4HB Immune Interaction Promotes Metastasis in Gastric Cancer Through Enhanced Cell Proliferation and Lipid Metabolism | [21] | Journal of Cellular and Molecular Medicine |
| **Abate M** | 2025 | Host Tissue Factors Predict Immune Surveillance and Therapeutic Outcomes in Gastric Cancer | [17] | Cancer Immunology Research |
| **Papadakos KS** | 2024 | The prognostic and potentially immunomodulatory role of cartilage oligomeric matrix protein in patients with gastric and esophageal adenocarcinoma | [41] | Cancer Immunology, Immunotherapy |
| **Yu H** | 2023 | CCR9-CCL25 mediated plasmacytoid dendritic cell homing and contributed the immunosuppressive microenvironment in gastric cancer | [20] | Translational Oncology |
| **Li X** | 2022 | Tumor-derived IL-8 facilitates lymph node metastasis of gastric cancer via PD-1 up-regulation in CD8+ T cells | [18] | Cancer Immunology, Immunotherapy |
| **Jiang H** | 2022 | Revealing the transcriptional heterogeneity of organ-specific metastasis in human gastric cancer using single-cell RNA Sequencing | [29] | Clinical and Translational Medicine |
| **Qian Y** | 2022 | Single-cell RNA-seq dissecting heterogeneity of tumor cells and comprehensive dynamics in tumor microenvironment during lymph nodes metastasis in gastric cancer | [27] | International Journal of Cancer |
| **Svensson MC** | 2021 | T cells, B cells, and PD-L1 expression in esophageal and gastric adenocarcinoma before and after neoadjuvant chemotherapy: relationship with histopathological response and survival | [22] | OncoImmunology |
| **Wang M** | 2021 | Lymph node metastasis-derived gastric cancer cells educate bone marrow-derived mesenchymal stem cells via YAP signaling activation by exosomal Wnt5a | * | Oncogene |
| **Donlon NE** | 2021 | The prognostic value of the lymph node in oesophageal adenocarcinoma; incorporating clinicopathological and immunological profiling | [19] | Cancers |
| **Dislich B** | 2017 | Expression patterns of programmed death-ligand 1 in esophageal adenocarcinomas: comparison between primary tumors and metastases. | [37] | Cancer immunology, immunotherapy: CII |

**References**

1.	Ouzzani M, Hammady H, Fedorowicz Z, Elmagarmid A (2016) Rayyan-a web and mobile app for systematic reviews. Syst Rev 5:210. <https://doi.org/10.1186/s13643-016-0384-4>

2.	The EndNote Team. EndNote. EndNote 2025 ed. Philidelphia, PA: Clarivate; 2013.

3.	Cochrane Handbook for Systematic Reviews of Interventions: Cochrane; 2024. Available from: [www.cochrane.org/handbook](https://sotonac-my.sharepoint.com/personal/jh9u24_soton_ac_uk/Documents/University/PhD/Review%20Article/www.cochrane.org/handbook).

4.	Page MJ, McKenzie JE, Bossuyt PM, Boutron I, Hoffmann TC, Mulrow CD, et al. (2021) The PRISMA 2020 statement: an updated guideline for reporting systematic reviews. BMJ 372:n71. <https://doi.org/10.1136/bmj.n71>
